# Extra-Thymic Physiological T Lineage Progenitor Activity Is Exclusively Confined to Cells Expressing either CD127, CD90, or High Levels of CD117

**DOI:** 10.1371/journal.pone.0030864

**Published:** 2012-02-15

**Authors:** Namita Saran, Jens Pommerencke, Katrin Witzlau, Malte Regelin, Andreas Krueger

**Affiliations:** Institute for Immunology, Hannover Medical School, Hannover, Germany; Oklahoma Medical Research Foundation, United States of America

## Abstract

T cell development depends on continuous recruitment of progenitors from bone marrow (BM) to the thymus via peripheral blood. However, both phenotype and functional characteristics of physiological T cell precursors remain ill-defined. Here, we characterized a putative CD135^+^CD27^+^ T cell progenitor population, which lacked expression of CD127, CD90, and high levels of CD117 and was therefore termed triple negative precursor (TNP). TNPs were present in both BM and blood and displayed robust T lineage potential, but virtually no myeloid or B lineage potential, *in vitro*. However, TNPs did not efficiently generate T lineage progeny after intravenous or intrathymic transfer, suggesting that a physiological thymic microenvironment does not optimally support T cell differentiation from TNPs. Thus, we propose that physiological T cell precursors are confined to populations expressing either CD127, CD90, or high levels of CD117 in addition to CD135 and CD27 and that TNPs may have other physiological functions.

## Introduction

T cell development depends on continuous recruitment of progenitor cells from bone marrow (BM) to the thymus via peripheral blood [1]. Thus, in order to qualify as physiological T cell precursors, cells a) must have T lineage potential; b) must be released into circulation; and c) must have the capacity to enter the thymus. Multiple populations have been described to fulfill all three criteria and some molecular cues required for progenitor entry into the thymus have been identified.

Using reductive isolation it has been demonstrated that all T cell precursors are confined to a heterogeneous population expressing CD135 (Flt3) and CD27 [2], [3]. However, it remains unclear which subsets contribute to intrathymic T cell development. The CD135^+^CD27^+^ population contains cells expressing high levels of CD117 (c-kit), that comprise various subsets of partially overlapping multipotent progenitors (MPP) that can be referred to as lymphoid-primed MPP (LMPP), early lymphoid progenitors (ELP) or L-selectin positive progenitors (LSP) [4], [5], [6]. These cells can give rise to both lymphoid and myeloid progeny, which is consistent with the notion that early T lineage progenitors retain myeloid potential inside the thymus even after losing B lineage potential [7], [8]. In addition, common lymphoid progenitors, characterized by expression of CD127 (IL-7Rα) and intermediate expression of CD117 are part of the CD135^+^CD27^+^ population. These cells are lymphoid-restricted *in vivo*. A third subset within the CD135^+^CD27^+^ population in BM expresses CD90 (Thy-1) in addition to low levels of CD117 and CD127. These cells may constitute direct precursors to circulating T lineage progenitors (CTP), which were identified in peripheral blood and are largely T lineage restricted *in vivo* [9]. Using lineage fate mapping based on the history of CD127 expression, which is indicative of lymphoid restriction, it has recently been suggested that the majority of early intrathymic precursors are derived from extrathymic cells that are already committed to the lymphoid lineage [10]. These data remain yet to be reconciled with the proposed contribution of T lineage precursors to intrathymic myeloid cells [11]. We have previously shown that multiple phenotypically distinct CD135^+^CD27^+^ progenitor subsets can contribute to T lineage differentiation in the thymus [3]. These subsets include cells expressing high levels of CD117, or are positive for CD127 or CD90. In addition, we observed low levels of transient T cell development even after depletion of all three subsets, suggesting that additional T lineage precursors with a CD117^−/low^CD127^−^CD90^−^ phenotype exist [3]. These cells expressed low levels of the chemokine receptors CCR7 and CCR9, which together are required for precursor entry into the thymus, as well as PSGL-1, the ligand for P-selectin, which also contributes to thymus seeding [12], [13], [14], [15].

Here we characterized lineage potential and T lineage differentiation of putative CD117^−/low^CD127^−^CD90^−^ T cell precursors *in vitro* and *in vivo* in order to test the hypothesis that non-MPP-like, non-CLP-like, non-CTP-like T cell precursors are present in BM and circulation.

## Results

### Characterization of putative CD117^−/low^CD127^−^CD90^−^ T cell precursors from BM and blood

**Figure 1:**
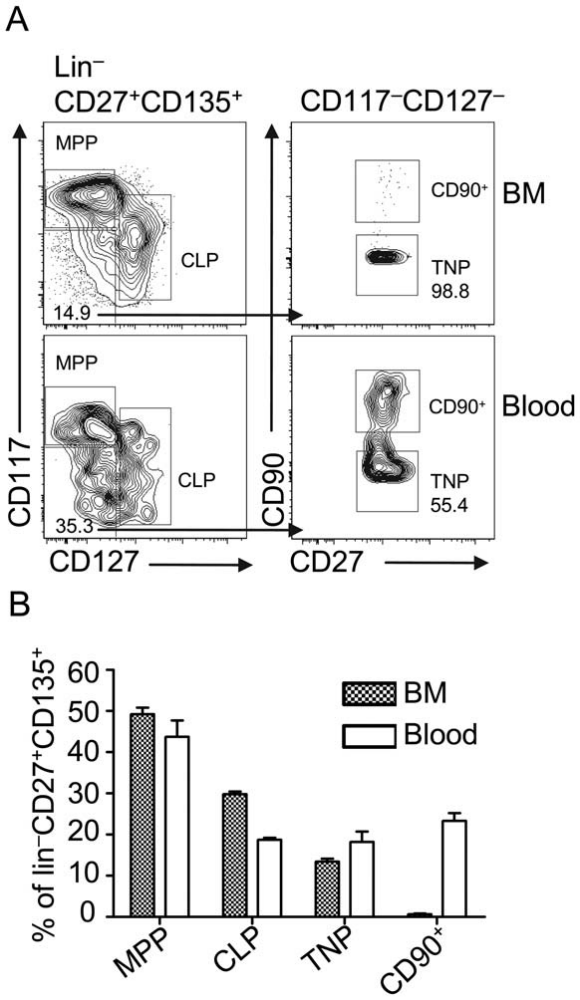
Characterization of TNPs from BM and blood. Lineage depleted BM and blood cells from C57BL/6 mice were stained for lineage markers, CD27, CD135, CD117, CD127 and CD90. A) Gating strategy for the identification of TNPs in BM and blood. Representative data from 1 out of 4 mice (BM) and pooled cells from 10 mice (blood). B) Relative quantification of MPPs (lin^−^CD27^+^CD135^+^CD127^−^CD117^hi^), CLPs (lin^−^CD27^+^CD135^+^CD127^+^CD117^+/low^), TNPs (lin^−^CD27^+^CD135^+^CD127^−^CD117^−/low^CD90^−^) and CD90^+^ (lin^−^CD27^+^CD135^+^CD127^−^CD117^−/low^CD90^+^) candidate T cell precursors within lin^−^CD27^+^CD135^+^ cells. Combined data of 4 mice and 20 mice for BM and blood, respectively, from 2 independent experiments. Error bars indicate SEM.

Multiple extrathymic T cell precursors expressing CD127, CD90, or high levels of CD117 have been characterized in BM and circulation. Using a competitive *in vivo* assay we have demonstrated that simultaneous depletion of CD117^hi^, CD127^+^ and CD90^+^ BM-derived precursor populations did not result in complete abrogation of T lineage reconstitution. This finding indicated the existence of an additional precursor population with a CD117^−/low^CD127^−^CD90^−^ surface marker profile, which we termed triple negative precursor (TNP) [3]. Prior to functional analysis we first determined the frequencies of this population in BM and circulation relative to other well defined T cell progenitors lacking markers of mature hematopoietic lineages (lin^−^) and being CD27^+^CD135^+^ (Figure 1A). In line with previous reports, CLPs (lin^−^CD27^+^CD135^+^CD127^+^CD117^+/low^) are less abundant in circulation than in BM, whereas the frequency of MPPs (lin^−^CD27^+^CD135^+^CD127^−^CD117^hi^) within lin^−^CD27^+^CD135^+^ cells was only slightly higher in BM when compared to blood (Figure 1B) [2], [16]. CD90^+^ cells were only present at low numbers in BM, but were present in blood at frequencies similar to MPPs [9]. The frequency of TNPs in BM was approximately half of the frequency of CLPs, while in blood the frequency of TNPs was comparable with that of CD90^+^ precursors (Figure 1B). Based on calculations made by us and others to determine absolute numbers of MPPs and CLPs in BM and blood we estimate that the observed frequencies of TNP correspond to 18,000 cells per femur and 50 cells per mL of blood [2], [3], [16]. It remains to be established though, whether TNPs constitute a homogeneous population. Nevertheless, TNPs are present in both BM and blood, thus fulfilling one critical characteristic of T cell progenitors.

### TNPs have robust T lineage potential

**Figure 2:**
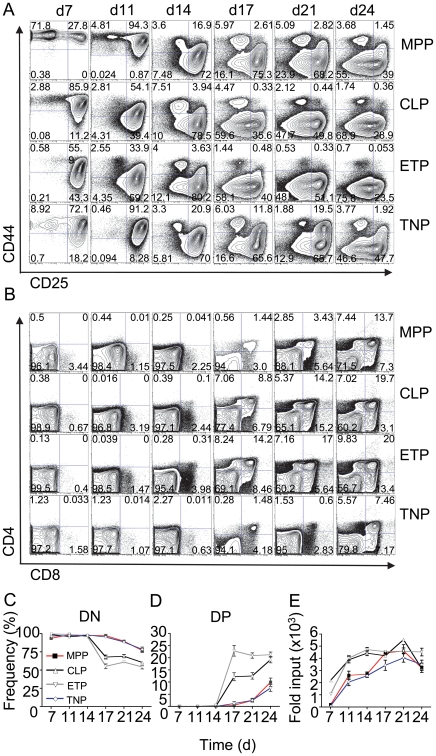
TNPs have robust T lineage potential. BM-derived MPPs, CLPs, TNPs and thymic ETPs were sorted from C57BL/6 mice and 500 cells of each population were cultured on OP9-DL1 cells in the presence of 1 ng/ml IL-7 and 5 ng/ml Flt3L. A) Cells were gated as CD4^−^CD8^−^ (DNs) and analyzed for expression of CD44 and CD25 at the indicated time points. One representative experiment out of 2 independent experiments is shown. B) Cells were analyzed for expression of CD4 and CD8 at the indicated time point. C, D) Quantification of data obtained from panel B. E) Relative expansion of cultures based on an input cell number of 500. Combined data of two independent experiments with 6 wells per experiment. Error bars indicate SEM.

The TNP frequency in BM was similar to that of other BM subsets with robust T lineage potential. To evaluate whether TNPs resemble canonical T cell progenitors and have T lineage potential, we cultured them under conditions that support T cell differentiation *in vitro* using OP9 murine stromal cells over-expressing the Notch ligand Dll-1 (OP9-DL1) [17]. As a control, other BM-derived precursors (MPPs and CLPs) and thymic early T cell progenitors (ETPs), which constitute the earliest canonical intrathymic progenitors identified to date, were analyzed as well. T cell differentiation kinetics were monitored for 24 days by assessing surface expression of CD44, CD25, CD4 and CD8 via flow cytometry every three to four days (Figure 2). MPPs showed slow kinetics at the start of the culture with a majority of cells still present at the CD44^+^CD25^−^ double-negative (DN) 1 stage at day 7, which by day 11 had proceeded further to the CD44^+^CD25^+^ DN2 stage (Figure 2A). At day 14 of culture the majority of MPPs had reached the CD44^−^CD25^+^ DN3 stage and continued to progress to the CD44^−^CD25^−^ DN4 stage starting at day 17. Concomitantly, at day 17 the first CD4^+^CD8^+^ double-positive (DP) cells became detectable (Figure 2B, D). CLPs and ETPs showed more rapid differentiation kinetics when compared to MPPs, giving rise to detectable amounts of DN3 cells already after 7 days of culture (Figure 2A, C). DN4 cells were detectable at day 14 of culture and low numbers of DP cells appeared at the same time (Figure 2B, D). After 17 days more than 10% and 20% DP cells were detectable in cultures derived from CLPs and ETPs, respectively (Figure 2B, C). The observed kinetics were in line with previously published data from us and others [18], [19].

Notably, TNPs displayed distinct differentiation kinetics from both MPPs and CLPs/ETPs. During the early phase of culture (until day 11) differentiation of TNPs paralleled that of CLPs/ETPs (Figure 2A, C, D). Subsequently, differentiation proceeded more slowly and DN4 cells as well as DP cells became detectable at day 17 of culture, similar to what we observed in MPP-derived cultures (Figure 2A, B). In addition, we assessed the expansion of cultures under T-promoting conditions, as robust T lineage potential is expected to be accompanied by considerable proliferation. All cultures started from various precursors expanded massively within the first 21 days of culture (Figure 2E). CLP-derived and ETP-derived cultures expanded more rapidly than MPP-derived cultures. Expansion of TNP-derived cultures largely paralleled that of MPPs, which is consistent with comparable differentiation kinetics during later stages of culture.

In conclusion, TNPs contain robust T lineage potential *in vitro*. Furthermore, it appears that T lineage differentiation in TNP-derived cultures is a biphasic process, which might indicate that the TNP population is not homogeneous.

### TNPs display limited non-T lineage potential

**Figure 3:**
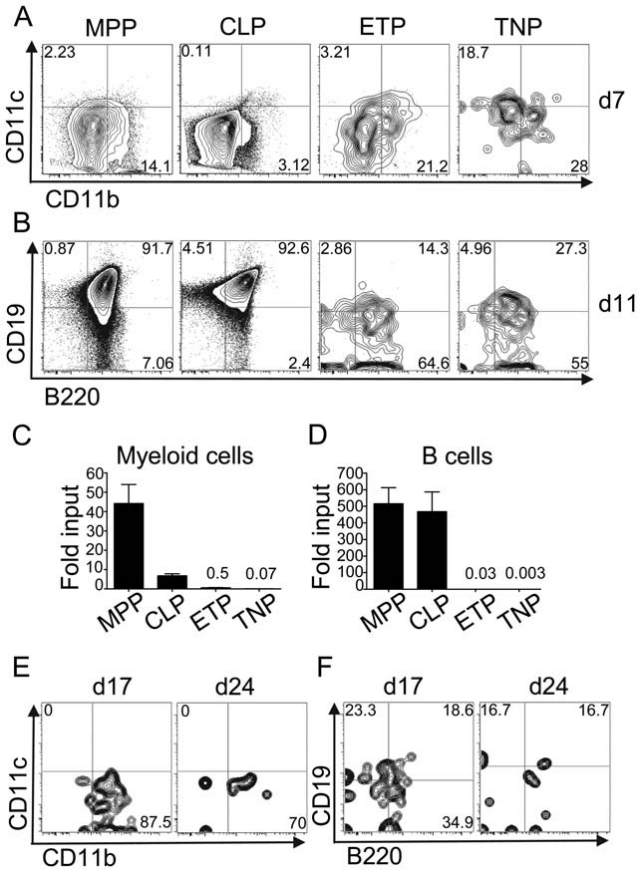
TNPs display limited non-T lineage potential. BM derived MPPs, CLPs, TNPs and thymic ETPs were sorted from C57BL/6 mice and 500 cells of each population were cultured on OP9 cells in the presence of 5 ng/ml IL-7 and 5 ng/ml Flt3L. A) Cells were analyzed for the expression of CD11c and CD11b at day 7 of culture. B) Cells were analyzed for the expression of CD19 and B220 at day 11 of culture. C) Relative expansion of myeloid cells after 7 days of culture (from 500 input cells). D) Relative expansion of B cells after 11 days of culture (from 500 input cells). C, D) Numbers above columns indicate fold expansion. E) TNP-derived cultures. Analysis was performed as in panel A) after 17 and 24 days. F) TNP-derived cultures. Analysis was performed as in panel B) after 17 and 24 days. A, B, E, F) One representative experiment out of 2 independent experiments is shown. C, D) Combined data of two independent experiments with 6 wells per experiment. Error bars indicate SEM.

T lineage precursors have been shown to display varying degrees of alternative lineage potential, ranging from being multipotent, via lymphoid restricted to largely T lineage committed. In order to investigate whether TNPs possessed alternative lineage potential for myeloid and B lineages, we cultured sorted ETPs, CLPs, MPPs and TNPs on OP9 murine stromal cells in the absence of the Notch ligand Dll-1. Myeloid potential was analyzed after 7 days of culture by staining for CD11c and CD11b (Figure 3A). Consistent with previous findings, both MPPs and ETPs generated high frequencies of CD11b^+^ cells, whereas the frequency of myeloid cells was very low in CLP-derived cultures [6], [10], [17]. Both, MPP-derived and CLP-derived cultures displayed massive expansion, resulting in the generation of substantial absolute numbers of myeloid cells in these cultures. Generation of myeloid cells from lymphoid precursors *in vitro* has recently been described [10], [20]. In cultures generated from TNPs, some CD11b^+^ cells could be detected, but they failed to expand under these conditions (Figure 3C). Consistently, few CD11b^+^ could be detected after 17 days of culture, but cultures were completely exhausted by day 24 (Figure 3E).

The generation of B lineage cells was assessed after 11 days of culture. Within this period of time, both MPPs and CLPs had generated substantial numbers of CD19^+^B220^+^ cells (Figure 3B, D). Neither ETPs nor TNPs gave rise to substantial amounts of CD19^+^ B lineage cells (Figure 3B). Furthermore, B lineage cells did not massively expand in these cultures (Figure 3D). In addition, we did not observe any B lineage cells after prolonged culture periods (Figure 3F).

In summary, these data indicate that TNPs harbor potential T cell precursors with limited or absent myeloid or B lineage potential *in vitro*.

### TNPs do not efficiently differentiate upon intravenous transfer

**Figure 4:**
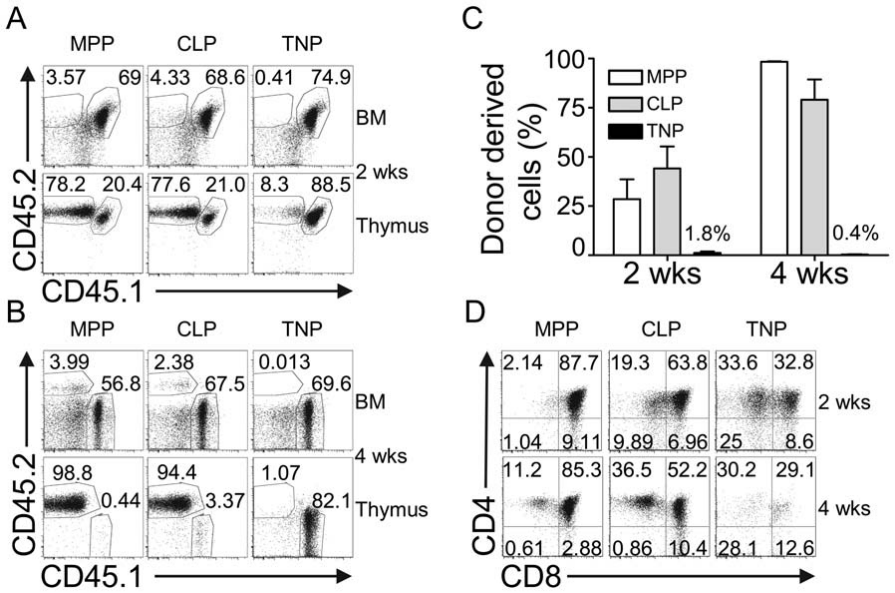
TNPs do not efficiently differentiate upon intravenous transfer. BM derived MPPs, CLPs and TNPs from C57BL/6 mice were sorted and 20,000 cells were intravenously injected into non-irradiated *Il7ra*-deficient mice expressing CD45.1 or CD45.1 and CD45.2. Donor derived cells were analyzed in thymus and BM by FACS for the expression of CD45.1 and CD45.2 at A) 2 weeks and B) 4 weeks after transfer. One representative experiment out of four and three (2 weeks and 4 weeks, respectively) independent experiments with 2 mice each is shown. C) Combined analysis of frequencies of donor-derived thymocytes of 4 and 3 individual experiments 2 and 4 weeks after transfer, respectively. Error bars indicate SEM. D) CD4 vs. CD8 surface expression of donor-derived thymocytes 2 and 4 weeks after transfer. One representative experiment out of four and three (2 weeks and 4 weeks, respectively) independent experiments with 2 mice each is shown.

Our findings indicated that TNPs are located both in BM and circulation and have robust T lineage potential, but limited alternative lineage potential, *in vitro*. Thus, they could constitute physiological T cell precursors, if they were able to seed the thymus *in vivo*. To test the capacity of TNPs to seed the thymus, we employed intravenous transfers into non-irradiated *Il7ra*-deficient hosts. Thymi of these mice have been previously shown to be receptive for T cell precursors independent of irradiation [21]. Donor-derived cells, which could be distinguished based on the expression of the congenic markers CD45.1 and CD45.2, were analyzed in BM and thymus 2 and 4 weeks after transfer. MPPs and CLPs were transferred as positive controls. Expectedly, at week 2 and 4 after transfer of MPPs or CLPs, thymocytes of donor origin (CD45.2^+^) were constituting the majority of cells (Figure 4A, B). In addition, some donor-derived cells were present in BM both after 2 and 4 weeks. Furthermore, CLP-derived and MPP-derived thymocytes had acquired CD4 and CD8 surface expression profiles consistent with normal developmental progression (Figure 4D).

Surprisingly, at 2 weeks after transfer TNP-derived donor thymocytes appeared only in two out of nine recipients and chimerism was low (Figure 4A, C). Furthermore, TNPs were absent from BM. Similarly, at 4 weeks after transfer only few, if any, thymocytes generated from TNPs were detectable (Figure 4B, C). Among donor-derived thymocytes, a large fraction was CD4 single positive, even after 2 weeks, and the frequency of DP cells was low when compared to thymocytes generated from CLPs or MPPs (Figure 4D). At 4 weeks after transfer the observed pattern of CD4 *vs.* CD8 expression was not markedly altered when compared to 2 weeks, suggesting that the DP thymocytes observed do not complete T cell development via the canonical SP stages. In order to exclude that TNP rapidly give rise to a short-lived wave of thymic T cell development, which is exported to the periphery, we also analyzed pooled lymph nodes for the presence of TNP-derived T cells. Approximately 40% donor-derived cells, virtually all of them B cells, could be detected in the periphery 2 weeks after transfer of either CLP or MPP (Figure S1). After 4 weeks both MPP and CLP had given rise to B cells and T cells and the frequencies of donor-derived cells amounted to 80% and 64%, respectively. In contrast, TNP-derived cells constituted only 1% and 3% in the periphery after 2 and 4 weeks, respectively. The majority of these cells were B cells, but very few peripheral T cells originating from TNP could be detected as well. These data suggest that TNP do not give rise to a short-lived wave of thymic T cell development resulting in rapid reconstitution of the periphery. Nevertheless, some TNP-derived B cells and few T cells were detectable in the periphery of *Il7ra*-deficient mice. As these mice are highly lymphopenic, even inefficiently generated B or T lineage progeny might result in detectable populations. Generation of B cells in this setting is consistent with the few B lineage cells observed in *in vitro* differentiation (Figure 3).

These results indicate that TNPs do not efficiently generate thymocytes after intravenous transfer. Non-canonical CD4 *vs.* CD8 profiles suggest that this might be due to a failure to enter the typical T lineage differentiation pathway *in vivo*.

### TNPs do not efficiently differentiate upon intrathymic transfer

**Figure 5:**
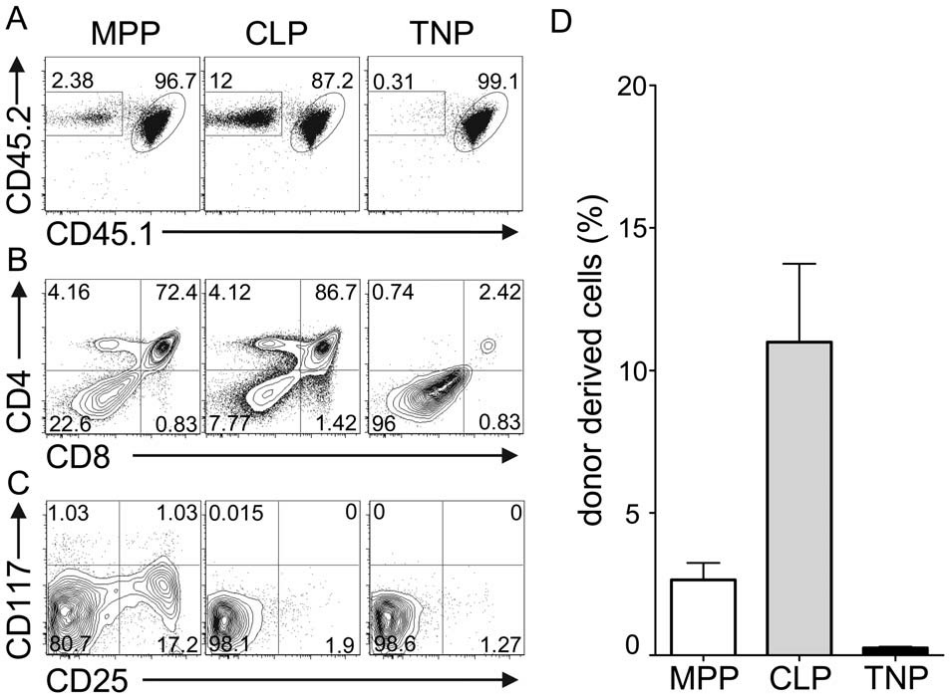
TNPs do not efficiently differentiate upon intrathymic transfer. BM derived MPPs, CLPs and TNPs from C57BL/6 mice were sorted and 20,000 cells were intrathymically injected into non-irradiated CD45.1 mice or CD45.1/2 mice. A) Donor derived cells were analyzed in thymus by FACS for the expression of CD45.1 and CD45.2 at 3 weeks after transfer. B) Donor derived cells from MPPs, CLPs and TNPs were also analyzed for CD4 and CD8 surface expression for identification of DN (CD4^−^CD8^−^), DP (CD4^+^CD8^+^) and SP (CD4^+^ or CD8^+^) cells. C) Donor derived electronically gated DN cells from MPPs, CLPs and TNPs were analyzed for CD117 and CD25 surface expression for identification of DN subsets (DN1: CD117^+^CD25^−^; DN2: CD117^+^CD25^+^; DN3: CD117^−^CD25^+^). One representative experiment out of three independent experiments with 3 mice each is shown. D) Combined analysis of 3 experiments with each 3 mice per group. Error bars indicate SEM.

TNPs displayed robust T lineage differentiation potential *in vitro* and also expanded massively under T-promoting conditions. Nevertheless, they failed to efficiently generate thymocytes *in vivo* upon intravenous transfer. This discrepancy could be based on defective homing of TNPs to the thymus, although they expressed low levels of CCR7, CCR9, and PSGL-1 [3]. Alternatively, *in vitro* culture may have provided differentiation and proliferation signals that are critical for T lineage differentiation of TNPs but are absent from the thymus. To test these possibilities we transferred various precursor cell populations directly into thymi of non-irradiated congenic wild-type recipients and analyzed recipient thymi 3 weeks after transfer for the presence of donor-derived cells. It should be noted that intrathymic transfer of precursor cells into non-irradiated wild-type mice is expected to result in lower frequencies of reconstitution than intravenous transfer into *Il7ra*-deficient recipients, as in the latter only donor-derived cells are receptive for IL-7 signals and will, thus, out-compete recipient cells. Whereas MPP-derived and CLP-derived thymocytes were readily detectable, only minute numbers of TNP-derived cells were identified in recipient thymi (Figure 5A, D). In contrast to the former cells, the latter had also failed to generate significant numbers of DP thymocytes (Figure 5B). However, generation of reduced numbers of DP cells could simply be due to a delay in differentiation as observed in vitro. Therefore, we also assessed DN subsets representing earlier differentiation stages. Whereas donor-derived DN1, DN2 and DN3 cells could still be detected in mice having received MPPs, none of these DN subsets were present among donor-derived cells after transfer of CLPs or TNPs (Figure 5C). Lack of CLP-derived DN subsets is consistent with fast differentiation kinetics. However, based on low numbers of DP cells TNPs would be predicted to differentiate with slower kinetics. Thus, in this case the absence of DN1 to DN3 cells indicates aberrant rather than fast differentiation. In addition, we analyzed some mice having received MPPs or TNPs intrathymically 5 weeks after transfer with essentially the same results as after 3 weeks. Percentages of TNP-derived thymocytes were very low when compared to MPP-derived cells. Furthermore, most cells had remained at the DN stage in the absence of DN1-DN3 cells. Similarly, very few TNP-derived T cells could be detected in the periphery (Figure S2). Thus, even after prolonged periods of time after intrathymic transfer TNPs do not yield substantial numbers of T lineage progeny.

Although these data do not rule out defective homing as a contributing factor for the very limited T lineage differentiation capacity of TNPs *in vivo*, these data clearly indicate that the thymic microenvironment fails to provide signals required for T lineage differentiation originating from TNPs.

## Discussion

Under physiological conditions intrathymic T cell precursors are not self-renewing. Thus, T cell development depends on continuous replacement of precursor cells from BM via peripheral blood. Here, we have characterized a putative T cell progenitor population, termed triple-negative precursor (TNP) based on its lack of expression of CD127, CD90 and high levels of CD117. We have previously shown that these cells express CD135 and CD27 and, thus, fulfill the minimal requirements for T cell precursors with regard to surface phenotype [2], [3]. Furthermore, TNPs are positive for CCR7, CCR9, and PSGL-1, all of which contribute to progenitor homing to the thymus [3], [13], [14], [15]. Together, it was therefore conceivable that these cells were genuine T cell progenitors. Here, we have extended the analysis of TNPs. We identified TNPs in peripheral blood and demonstrated their robust T lineage potential *in vitro*. Concomitantly, TNPs virtually lacked myeloid potential as well as B lineage potential. Nevertheless, TNPs failed to consistently generate T lineage cells *in vivo* after intravenous or intrathymic application, indicating that these cells are inefficient T lineage progenitors.

TNPs may be comprised of several different populations. This notion was supported by the observation of biphasic T lineage differentiation kinetics of TNPs *in vitro*. One population that might be part of TNPs are CLP-like cells lacking substantial surface expression of CD127 [22]. However, in contrast to TNPs, CLP-like cells possessed robust B lineage potential, thus indicating that these cells are not contained within TNPs. CLP-like cells express CD135, but expression of CD27 has not been assessed. As TNPs should comprise all CD117^−/low^CD127^−^CD90^−^ precursors our data implicate that CD127^−^ CLP-like cells do not constitute physiological T cell precursors.

TNPs displayed little non-T lineage potential *in vitro* in OP9 co-cultures. Although T lineage specification and commitment are generally assumed to be intrathymic events elicited by Notch signaling, various prethymic T lineage restricted progenitor populations have been described. Thus, we have identified a rare T-lineage committed progenitor population in circulation (CTP), which expressed CD90 and could be generated in the absence of a thymus or Notch signals [9]. Similar populations sharing expression of CD90 were also identified in BM and spleen [23], [24]. However, precursor-progeny relationships have not been clearly established between the different CD90^+^ T cell precursors.

To our surprise TNPs did not efficiently generate T cells *in vivo*. As neither intravenous nor intrathymic application resulted in substantial T cell progeny, we could not directly address the homing capacity of TNPs to the thymus. However, we have reported previously that TNPs are positive for CCR7, CCR9 as well as PSGL-1, thus expressing a fundamental set of thymus homing receptors [3], [12], [13], [14]. Failure to give rise to T cells upon intrathymic transfer despite robust T lineage potential *in vitro* indicates that TNPs require factors for T lineage differentiation that are not supplied by the thymus under physiological conditions, but are present in OP9-DL1 cultures. Given that TNPs are negative for or express very low amounts of CD127, the receptor for IL-7, it is possible that the intrathymic concentration of IL-7 is not sufficient to promote survival of TNPs. Similarly, other cytokines, such as SCF, the ligand for CD117, might not be present at sufficient amounts in the normal thymus to stimulate TNPs that display low receptor densities.

If TNPs do not represent efficient T lineage precursors in vivo, is there anything to be learned from this study? First, our data provide additional minimal surface characteristics to qualify T cell precursors. Thus, in addition to CD135 and CD27, genuine T cell progenitors can be identified by expression of either CD127, CD90, or high levels of CD117. Our data suggest that not all bona fide progenitor cells, which fulfill the minimal requirements to qualify as T cell progenitors, i.e. T lineage potential, presence in BM and circulation, and capacity to seed the thymus (represented by expression of the relevant homing receptors), do contribute to T cell development. Second, it was consistently possible to differentiate TNPs *in vitro* to generate bona fide DN3 and even DP thymocytes. Therefore, TNPs may serve as a source of cells, which, after *in vitro* differentiation and expansion, could be used for T lineage generation. Such *in vitro* generated thymocytes have been applied earlier in immune regeneration as well as a source of alloreactive T cells for tumor therapy [25], [26]. Third, T lineage potential has recently been described in preGM cells, a subset of common myeloid progenitors. Although these cells lacked the capacity to seed the thymus, probably because they do not express CCR7 and CCR9, they underwent malignant transformation upon constitutive activation of Notch signaling and gave rise to T cell leukemia [27]. Thus, also inefficient or non-physiological T cell progenitors, such as TNPs, might constitute the source of malignancies of the T lineage.

## Materials and Methods

### Ethics statement

All animal experiments were carried out according to institutional guidelines approved by the Niedersächsisches Landesamt für Verbraucherschutz und Lebensmittelsicherheit. This study was approved under permit number 33.9-42502-04-07/1393.

### Mice

*Il7ra*-deficient mice (B6.129S7-*Il7r^tm1Imx^*/J) and B6.SJL-*Ptprc^a^ Pepc^b^*/BoyJ mice (C57BL/6 mice carrying the CD45.1 allele, termed “B6 CD45.1” throughout this manuscript) were purchased from Jackson Laboratories (Bar Harbor, ME, U.S.A.). C57BL/6J mice (CD45.2), *Il7ra*-deficient mice expressing CD45.1 or CD45.1 and CD45.2 were bred at the animal facility of Hannover Medical School. Animals were maintained under specific-pathogen-free conditions.

### Antibodies and flow cytometry

Monoclonal antibodies specific for CD4 (RM4-5, GK1.5), CD8 (53-6.7), CD25 (PC61), CD44 (IM7), Gr-1 (RB6-8C5), erythroid cell marker (Ter-119), CD19 (1D3), CD11b (M1/70), CD11c (N418), pan-NK (DX5), CD45.1 (A20), CD45.2, B220 (RA3-6B2), CD117 (2B8, ACK2), Sca-1 (E13-161.7), CD90.2 (53-2.1), CD135 (A2F10), CD127 (A7R34), CD27 (LG.3A10), TCRβ (H57-597) were used either as purified or as biotin, PacificBlue, eFluor450, fluorescein isothiocyanate (FITC), Alexa488, phycoerythrin (PE), peridinin chlorophyll protein-Cy5.5 (PerCP-Cy5.5), PE-Cy7, allophycocyanin (APC), APC-Cy7, or APC-Alexa750 conjugates. Antibodies were purified from hybridoma supernatants using standard procedures or were purchased from eBioscience, BD Biosciences or Biolegend. PE-Cy7 conjugated streptavidin (BD Biosciences) was used to reveal staining with biotinylated mAb. Flow cytometric analysis was performed on a BD LSRII (BD, San Jose, CA). Data were analyzed with FlowJo software (Treestar). For analysis, dead cells and debris were excluded by appropriate gating of forward and sideward scatter. Lineage negative cells were isolated from total BM by staining cell suspensions with a lineage-specific antibody cocktail (anti-CD4, anti-CD8, anti-CD19, anti-CD11b, anti-Gr-1, Ter-119, and DX5) followed by incubation with anti-rat-IgG-conjugated magnetic beads (Dynal, Invitrogen) and magnetic bead depletion of mature lineages. Enriched cell suspensions were surface stained with lineage markers conjugated to PE-Cy7. Isolation of blood cells was performed as previously described [9]. For isolation of ETPs double negative thymocytes were enriched by complement lysis. Thymocyte suspensions were labelled with anti-CD4 (RL172.1) and anti-CD8 (M31) antibodies and subsequently subjected to lysis using LowTox rabbit complement (Cedarlane). Life cells were recovered on a Lympholyte M gradient (Cedarlane) and stained with antibodies against lineage markers (CD4, CD8, CD19, NK1.1), CD117, CD44, and CD25. Cells were double-sorted using a FACSAria (BD); sorted cells were of ≥95% purity.

### Intravenous adoptive transfers

Lin^−^ BM cells were prepared as described above. 20×10^3^ MPPs, CLPs, TNPs from BM were sorted from CD45.2 mice. In some experiments donor cells were CD45.1/CD45.2 heterozygous to exclude potential recipient-derived effects or possible effects due to different CD45 alleles. No significant recipient-derived effects or variances due to different CD45 alleles were observed. Donor cells were transferred intravenously into non-irradiated *Il7ra*-deficient hosts, which allow continuous thymus seeding, while maintaining an intact thymic architecture. Thymi, BM, and pooled lymph nodes (peripheral and mesenteric lymph nodes) were analyzed flow cytometrically 2 and 4 weeks after transfer.

### Intrathymic transfers

20×10^3^ MPPs or CLPs or TNPs isolated from B6 CD45.2 mice were injected into thymi of non-irradiated B6 CD45.1 mice. Thymi and pooled lymph nodes (peripheral and mesenteric lymph nodes) were analyzed for donor-derived cells 21 or 35 days after transfer.

### OP9 co-cultures

OP9-DL1/OP9-GFP co-culture assays were essentially performed as described [17]. Sorted MPPs, CLPs, ETPs or TNPs from CD45.2 mice were plated onto subconfluent OP9-DL1/OP9-GFP monolayers at 500 cells/well in a 24 well plate. Co-cultures were performed in the presence of 1 ng/ml IL-7 and 5 ng/ml Flt3 ligand (Flt3L). At day 4 of differentiation the culture medium was exchanged and at day 7 thymocytes were seeded onto fresh OP9-DL1/OP9-GFP monolayers and lineage differentiation were then checked for 24 days.

### Statistical analysis

All analysis was performed using GraphPad Prism software. Data are represented as mean ± SEM.

## Supporting Information

Figure S1 **TNPs do not reconstitute T cells in lymph nodes.** Lymph nodes from mice described in Figure 4 were analyzed flow cytometrically for donor-derived T cells. Frequencies of donor-derived cells for each condition are shown above dot plots. Dot plots show electronically gated donor-derived cells (CD45.2^+^CD45.1^−^). Numbers in dot plots indicate percentages of T cells among donor-derived cells. N = 2–4 mice per group.
(TIF)

Figure S2 **TNPs do not efficiently differentiate within prolonged periods of time upon intrathymic transfer.** Experimental setup as in Figure 5. Mice were analyzed 5 weeks after transfer. A) Frequency of donor-derived cells 5 weeks after transfer of MPPs or TNPs. B) Phenotype of donor-derived thymocytes after 5 weeks. Left panels: CD4 vs. CD8 plots; right panels CD117 vs. CD25 plots of electronically gated DN cells. C) Donor-derived cells in lymph nodes. A) Each dot represents an individual mouse. B, C) Representative plots of three mice analyzed.
(TIF)
