# Supplementary figures and images for: Extra-Thymic Physiological T Lineage Progenitor Activity Is Exclusively Confined to Cells Expressing either CD127, CD90, or High Levels of CD117
Source: PLoS One. 2012 Feb 15;7(2):e30864. doi: 10.1371/journal.pone.0030864 (PMC3280270; doi:10.1371/journal.pone.0030864)

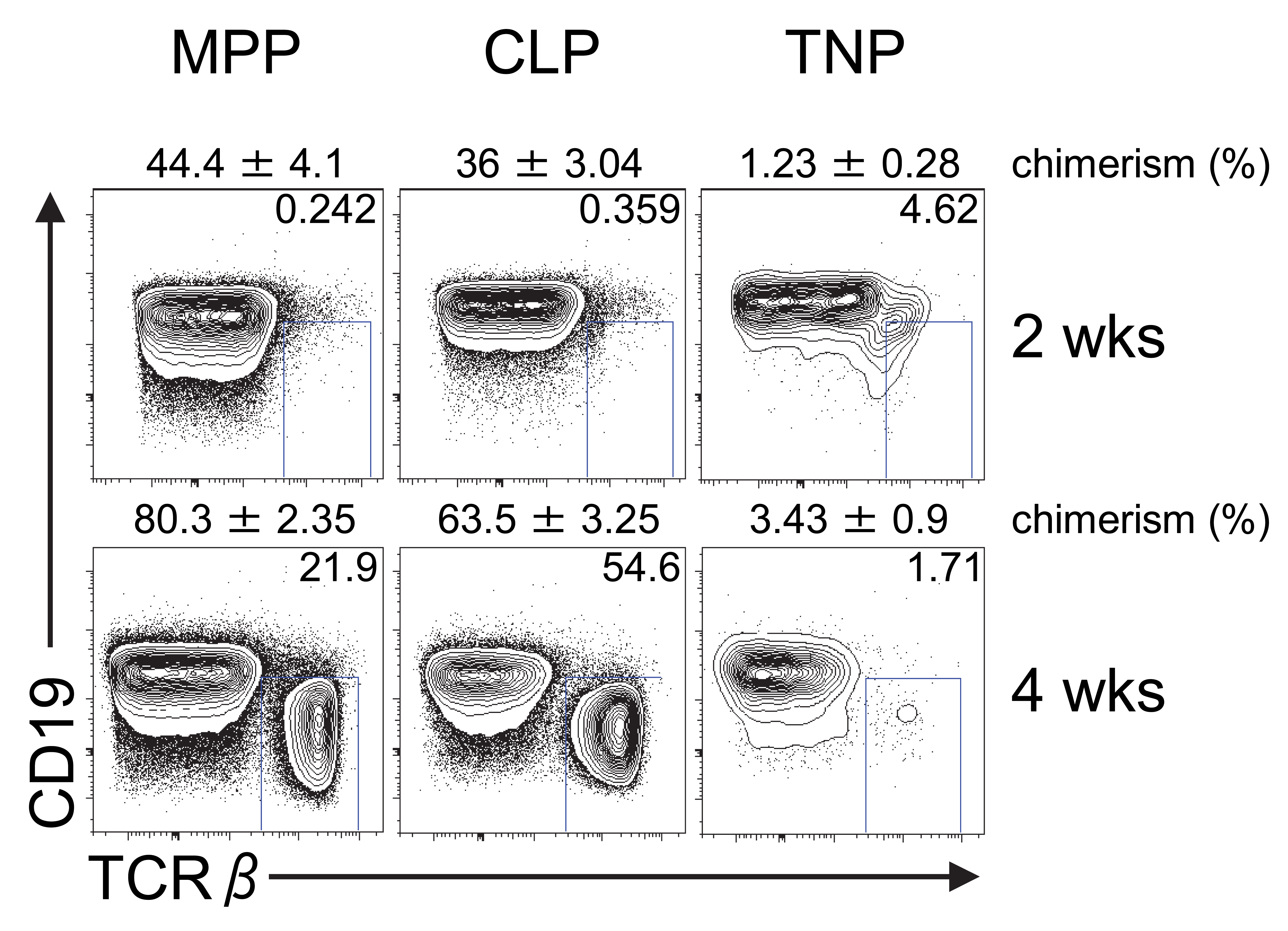

Supplement: Figure S1 — TNPs do not reconstitute T cells in lymph nodes. Lymph nodes from mice described in Figure 4 were analyzed flow cytometrically for donor-derived T cells. Frequencies of donor-derived cells for each condition are shown above dot plots. Dot plots show electronically gated donor-derived cells (CD45.2+CD45.1−). Numbers in dot plots indicate percentages of T cells among donor-derived cells. N = 2–4 mice per group. (TIF) [file pone.0030864.s001.tif]

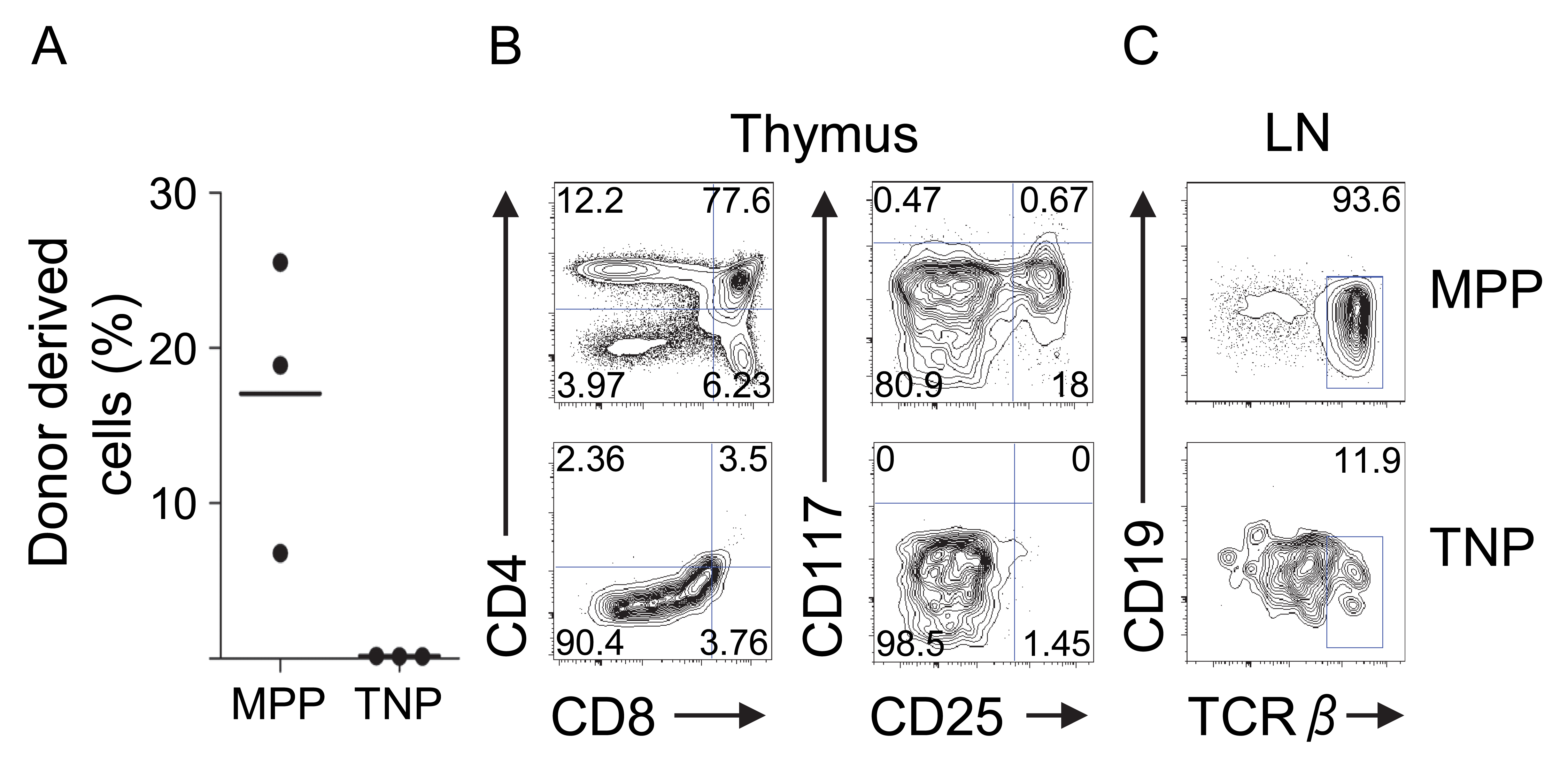

Supplement: Figure S2 — TNPs do not efficiently differentiate within prolonged periods of time upon intrathymic transfer. Experimental setup as in Figure 5. Mice were analyzed 5 weeks after transfer. A) Frequency of donor-derived cells 5 weeks after transfer of MPPs or TNPs. B) Phenotype of donor-derived thymocytes after 5 weeks. Left panels: CD4 vs. CD8 plots; right panels CD117 vs. CD25 plots of electronically gated DN cells. C) Donor-derived cells in lymph nodes. A) Each dot represents an individual mouse. B, C) Representative plots of three mice analyzed. (TIF) [file pone.0030864.s002.tif]
